# Active open-loop control of elastic turbulence

**DOI:** 10.1038/s41598-020-72402-y

**Published:** 2020-09-24

**Authors:** Reinier van Buel, Holger Stark

**Affiliations:** Institute of Theoretical Physics, Technische Universität Berlin, Hardenbergstrasse 36, 10623 Berlin, Germany

**Keywords:** Fluid dynamics, Nonlinear phenomena, Phase transitions and critical phenomena

## Abstract

We demonstrate through numerical solutions of the Oldroyd-B model in a two-dimensional Taylor–Couette geometry that the onset of elastic turbulence in a viscoelastic fluid can be controlled by imposed shear-rate modulations, one form of active open-loop control. Slow modulations display rich and complex behavior where elastic turbulence is still present, while it vanishes for fast modulations and a laminar response with the Taylor–Couette base flow is recovered. We find that the transition from the laminar to the turbulent state is supercritical and occurs at a critical Deborah number. In the state diagram of both control parameters, Weissenberg versus Deborah number, we identify the region of elastic turbulence. We also quantify the transition by the flow resistance, for which we derive an analytic expression in the laminar regime within the linear Oldroyd-B model. Finally, we provide an approximation for the transition line in the state diagram introducing an effective critical Weissenberg number in comparison to constant shear. Deviations from the numerical result indicate that the physics behind the observed laminar-to-turbulent transition is more complex under time-modulated shear flow.

## Introduction

Controlling the flow pattern of viscoelastic fluids is extremely challenging due to their inherent non-linear properties and their strong response to shear deformations^1–3^. Viscoelastic fluids, such as polymer solutions, exhibit transitions from steady to time-dependent non-laminar flows, which is useful for heat and mass transport at the micron scale^1,2,4–9^ whereas in Newtonian fluids transport on such small scales is dominated by diffusion. Turbulent viscoelastic flow fields show similar properties as their counterparts in Newtonian fluids^4^. Consequently, the state of the occurring flow pattern is called *elastic turbulence*^1^. Since the discovery of this seminal effect at the beginning of the new millennium^1^, research is ongoing^10–16^. The transition to elastic turbulence is accompanied by an enhanced drag resistance in flowing polymer solutions^1,2,4,15^. In this work we report on the rich complex behaviour initiated in viscoelastic flows by applying an active open-loop control scheme in the form of a time-modulated shear rate. This method reduces and ultimately suppresses elastic turbulence.

Active or dynamic control requires auxiliary energy, while passive or static control requires none^17^. Both control schemes applied to flow patterns and fluid instabilities in Newtonian fluids have extensively been studied^18–25^. Also, passive control of viscoelastic fluid flow has been examined^26–31^ using either geometric modifications^28,29^ including spatially modulated cylinders in a Taylor–Couette geometry^30^ and disorder in microfluidic flows to inhibit elastic turbulence^31^, or soft boundaries^27^, as well as thermal control^26^. In contrast, the search for active control strategies appropriate for viscoelastic fluids has so far been limited. Different responses of a Poiseuille flow to periodically modulated driving were observed^32^, while additional axial flow in a Taylor-Couette geometry delays the onset of the elastic instability^33^.

In Newtonian fluids the transition to turbulence is solely driven by inertia and therefore characterized by the Reynolds number Re^34^. In the following we concentrate on small Reynolds numbers, where inertia can be neglected. Then the transition from steady laminar flow to elastic turbulence is determined by the Weissenberg number $\mathrm {Wi}$, the product of an intrinsic fluid relaxation time and the fluid deformation rate^35,36^.

Importantly, the critical Weissenberg number, at which this transition occurs, depends on the geometry and the curvature of the stationary flow streamlines^35^. In experiments with curved streamlines a purely elastic instability has been observed in Taylor–Couette flow^4,37^, von Kármán swirling flow^1,4,38–40^, serpentine channel or Dean flow^4,41^, cone-and-plate flow^38^, cross-channel flow^9,42^, and lid-driven cavity flows^35^. Viscoelastic fluids flowing through straight microchannels are linearly stable and non-linearly unstable^43–45^. Different numerical techniques were employed to solve equations modeling viscoelastic fluids and thereby also revealed the purely elastic instability in similar geometries. Articles address unbounded flows with sinusoidal forcing^6,46,47^, Kolmogorov flow^48,49^, as well as wall-bounded flows, including sudden-expansion flow^50^, channels with cross-slot geometry^51^ or serpentines^52^ and the Taylor–Couette geometry^53–55^. Thus, demonstrating the importance of numerical techniques in understanding the underlying physical principles in complex fluid flows.

Here, we realize active open-loop control, meaning control without feedback, by modulating the applied shear rate in time. We characterize the modulation through the Deborah number $\mathrm {De}$, the ratio of the intrinsic fluid relaxation time to the typical time of deformation. Hence, De describes the degree of elastic response to an external forcing applied over a given time frame^36,56^.

We present first results on actively controlling elastic turbulence in a viscoelastic fluid and thereby provide an important step towards applying further active control strategies to viscoelastic fluids. We obtain numerical solutions of the Oldroyd-B model in a 2D Taylor–Couette geometry. We explicitly choose the Oldroyd-B model for its simplicity to reduce the number of free parameters and have no shear dependency on the viscosity. Since our analysis is restricted to two spatial dimensions, we can provide a thorough and careful analysis of how elastic turbulence is reduced and ultimately vanishes by tuning the oscillation frequency in the simplest implementation of a wall-bounded flow. Although our setting does not access the three dimensions of experimental flows, we can gain general insight into controlling viscoelastic fluids and their response to time-dependent shear. We have shown an elastic instability towards elastic turbulence at $\mathrm {Wi}=10$ in earlier work, where we applied a shear rate constant in time in the same geometry^55^. In this work, we use two kinds of time-modulated shear rates in the form of a square or sine wave, which display similar results. We demonstrate how elastic turbulence is significantly reduced with increasing modulation frequency and ultimately vanishes at a critical Deborah number $\mathrm {De}_c$. Here, the flow field assumes the radially symmetric base flow of the non-turbulent case.

## Results

**Figure 1:**
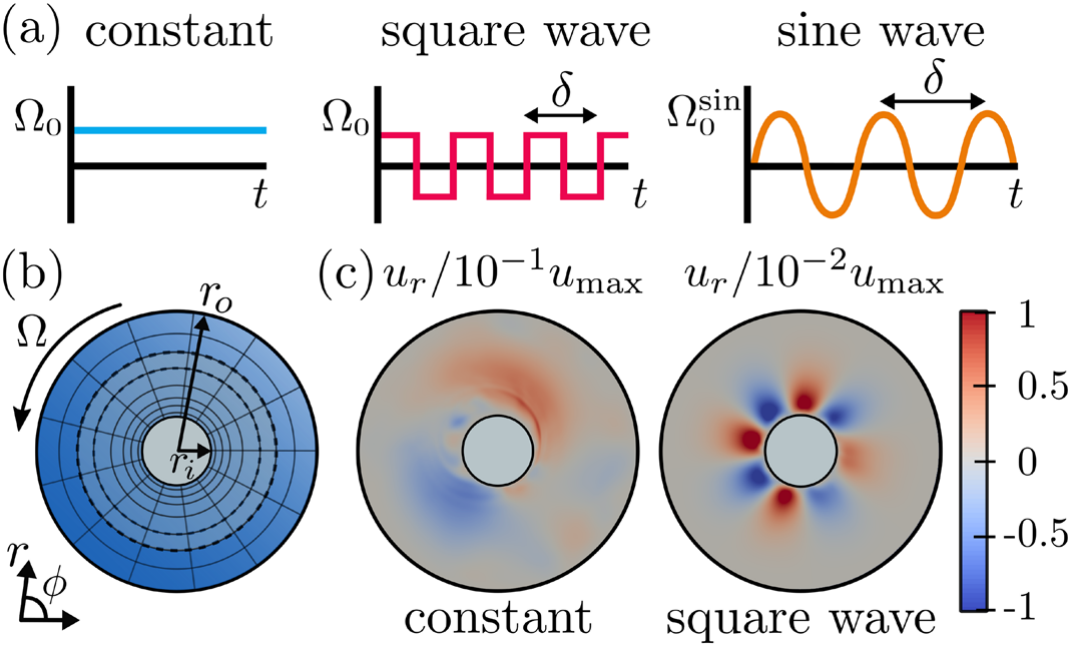
(**a**) Angular velocity ${\Omega }$ versus time of the outer cylinder for different driving protocols. (**b**) Schematic of the 2D Taylor–Couette geometry. The outer cylinder rotates with angular velocity $\Omega$. (**c**) Color-coded radial velocity field component $u_r$ normalized by the maximum velocity $u_\text {max}$ for $\mathrm {Wi} = 21.4$. Left: at time $t=225$, where $\Omega$ is constant. Right: at $t=375$ after the square-wave modulated driving with $\mathrm {De}=0.28$ has been switched on.

We examine the flow field ${\varvec{u}}({\varvec{r}},t)$ of an incompressible viscoelastic fluid in a 2D Taylor–Couette geometry. The inner cylinder, at radius $r_i$, is fixed and the outer cylinder, at radius $r_o=4\; r_i$, rotates with a periodically modulated angular velocity $\Omega$ with period $\delta$, see Fig. 1b. We distinguish between two modulations: a square wave with amplitude $\Omega _0$ and a sine wave with amplitude $\Omega _0^\text {sin}$, see Fig. 1a. These amplitudes are chosen such that the Weissenberg numbers defined with $|\Omega |$ averaged over one period are equal, $\mathrm {Wi}=1/\delta \int _{0}^{\delta } \lambda |{\Omega }|\mathrm {d}t = \lambda \Omega _0$, with $\lambda$ the elastic relaxation time of the fluid. The Deborah number is determined by the rate of change in the shear flow and thus is given by $\mathrm {De}= \lambda / \delta$. We use dimensionless quantities and rescale lengths by $r_o$ and time by $2\pi \Omega ^{-1}$.

To model the viscoelastic fluid, we use the Oldroyd-B model. It uses the constitutive relation for the polymeric stress tensor,

1 $$\begin{aligned} {\varvec{\tau }} +{\mathrm{Wi}\,} \overset{\nabla}{{\varvec{\tau}}} = {\beta} \left[ \nabla \otimes {\mathbf{u}} + (\nabla \otimes {\mathbf{u}})^{\mathrm{T}} \right] \, , \end{aligned}$$

where $\beta =\eta _p/\eta _s$ is the ratio of the polymeric and solvent shear viscosities, and $\overset{\nabla }{{\varvec{\tau }}}$ denotes the upper convective derivative of ${\varvec{\tau }}$ defined as

2 $$\begin{aligned} \overset{\nabla}{{\varvec{\tau}}} = \frac{\partial {\varvec{\tau}}}{\partial t} + \left( {\mathbf{u}}\cdot \nabla \right) {\varvec{\tau }} - (\nabla \otimes {\mathbf{u}})^{\mathrm{T}}{\varvec{\tau}} - {\varvec{\tau}} ( \nabla \otimes {\mathbf{u}}) \, . \end{aligned}$$

The hydrodynamic continuity equations for density $\rho$ and momentum read

3 $$\begin{aligned} \nabla \cdot \mathbf {u}&= 0 \, , \end{aligned}$$

4 $$\begin{aligned} \mathrm {Re} \left[ \frac{\partial \mathbf {u}}{\partial t} + \left( \mathbf {u} \cdot \nabla \right) \mathbf {u} \right]&= - \nabla p + \nabla ^2 \mathbf {u} + \nabla {\varvec{\tau }} \, . \end{aligned}$$

We always set the Reynolds number $\mathrm {Re} = \rho {\Omega _0} r_o^2 / \eta _s\approx 10^{-4}$ such that fluid inertia is negligible.

We obtain numerical solutions of the model equations using the open-source program OpenFOAM^®^. Further details are presented in the Methods section. The simulations with a time-modulated driving of the outer cylinder are either started from rest (velocity and stress tensor fields are zero) or from a turbulent state, which is obtained starting with a constant shear rate $\Omega _0$ for the first 250 rotations. The flow field is characterized through its fluctuations relative to the base flow $\mathbf {u}^0(\mathbf {r},t)=u_\phi ^0 \mathbf {e}_\phi$ by the secondary-flow strength

5 $$\begin{aligned} \sigma (t) \equiv \sqrt{\left\langle [\mathbf {u}(\mathbf {r},t)-\mathbf {u}^0(\mathbf {r}, {t})]^2 \right\rangle _{r,{\phi }}} \big / u^0_{\mathrm {max}} \, . \end{aligned}$$

The azimuthal base-flow component is $u_\phi ^0 = A r + B r^{-1}$, $A = \frac{r^2_o }{r^2_o-r^2_i}\Omega$, and $B = -\frac{r^2_i r^2_o}{r^2_o-r^2_i}\Omega$^37^, where for $\Omega$ we take the periodically modulated angular velocity. It solves the Oldroyd-B model in the laminar case without turbulence. Here, $\langle ...\rangle _{r,\phi }$ denotes the spatial average over coordinates $r,\phi$. For constant $\Omega$ and $\mathrm {Wi}>\mathrm {Wi}_c=10$, the secondary-flow strength is increasingly irregular with increasing $\mathrm {Wi}$. The radial symmetry of the flow is broken, a radial velocity component $u_r(r,\phi )$ emerges [see Fig. 1c, left, and Movie S1], and a supercritical transition is observed^55^. We add a remark here. We did not observe a multi-stage transition from laminar to turbulent flow neither in^55^ nor in this work as might be expected similar to viscous fluids. While several states are reported in the inertial regime of the elastic instability both in experiments^57,58^ and theory^53^, at $\mathrm {Re} \ll 1$ only disordered oscillations are observed in the experiments, which can be closely associated with elastic turbulence^4,53^. Furthermore, the linear stability analysis of the Oldroyd-B model for axisymmetric modes in Ref.^59^ demonstrates that a broad band of wavelengths becomes unstable for small increases in the Weissenberg number. Similar behavior could be the reason that a multi-stage transition cannot be resolved in our simulations.

**Figure 2:**
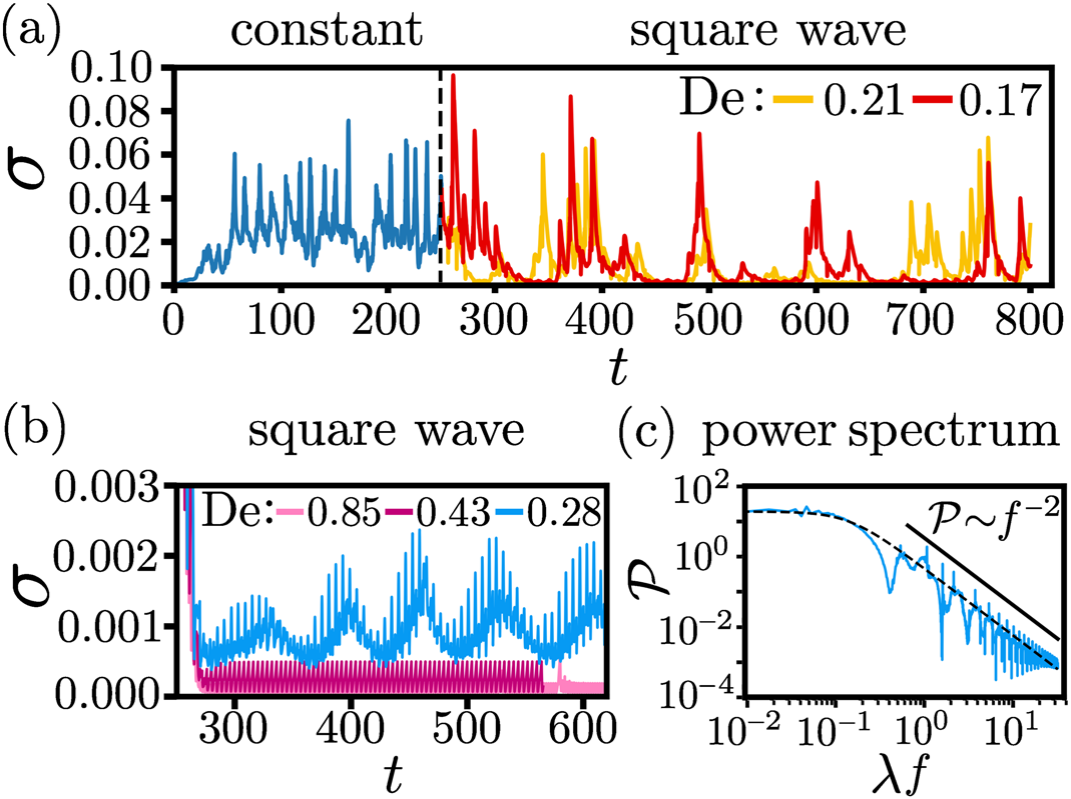
(**a**, **b**) Secondary-flow strength $\sigma$ as a function of time *t*. The outer cylinder rotates with a constant angular velocity $\Omega _0$ for the first 250 rotations. Then the modulated square-wave driving with amplitude $\Omega _0$ and different $\mathrm {De}$ is switched on. The Weissenberg number is $\mathrm {Wi}=21.4$. (**c**) Temporal power spectrum of $\sigma$, $\mathscr {P}=|\mathscr {F}(\sigma )|^2$, for $\mathrm {De}=0.28$. The observed spectrum scales as $\mathscr {P}\sim f^{-2}$ and the dotted line is a Lorentzian fit, given by Eq. (6), with $\lambda f_c = 0.15$.

The secondary-flow strength $\sigma$ can be significantly lowered by applying a square-wave driving to the outer cylinder as a comparison to the case of constant rotational velocity shows in Fig. 2 [see also Fig. 1c, right, and Movie S2]. For Weissenberg number $\mathrm {Wi}=21.4$ we present $\sigma$ for driving frequencies in the range $0.17 \le \mathrm {De} \le 0.85$ and observe that it decreases with increasing $\mathrm {De}$. For lower frequencies ($\mathrm {De}=0.21$ or $\mathrm {De}=0.17$) $\sigma$ exhibits irregular peaks in time, which have magnitudes comparable to the case of constant rotation (also see Movie S3). However, in between the irregular peaks the magnitude of $\sigma$ is much smaller and fluctuations in the flow are suppressed. For high frequencies $\sigma$ strongly tends to zero. It shows oscillations with a period equal to the driving period $\delta$ [see Fig. 2b], before the flow ultimately becomes laminar. Moreover, at $\mathrm {De}=0.28$, the amplitude of the fast oscillations of the secondary-flow strength seems to be modulated periodically. However, the power spectrum of $\sigma (t)$ does not reveal such a regular modulation. It can be well fitted by a Lorentzian

6 $$\begin{aligned} {\mathscr {P} \sim \frac{1}{1 + (\lambda f / \lambda f_c)^2}\, , } \end{aligned}$$

which corresponds to an exponential decay of the auto-correlation function of the order parameter, $\langle \sigma (0) \sigma (t) \rangle$. The critical frequency $f_c$ is directly related to the decay time $t_c = (2\pi f_c )^{-1}$ of the autocorrelations and from Fig. 2c we roughly find $t_c / \lambda = (2\pi \lambda f_c )^{-1} \approx (2 \pi \cdot 0.15 )^{-1} = 1.06$, *i.e.*, $t_c$ is close to $\lambda$. Superimposed on the power-law decay of $\mathscr {P}$ are peaks at frequencies, which are multiples of $\delta ^{-1}$ as a closer insprection of Fig. 2c shows. They correspond to the fast modulations seen in $\sigma (t)$.

**Figure 3:**
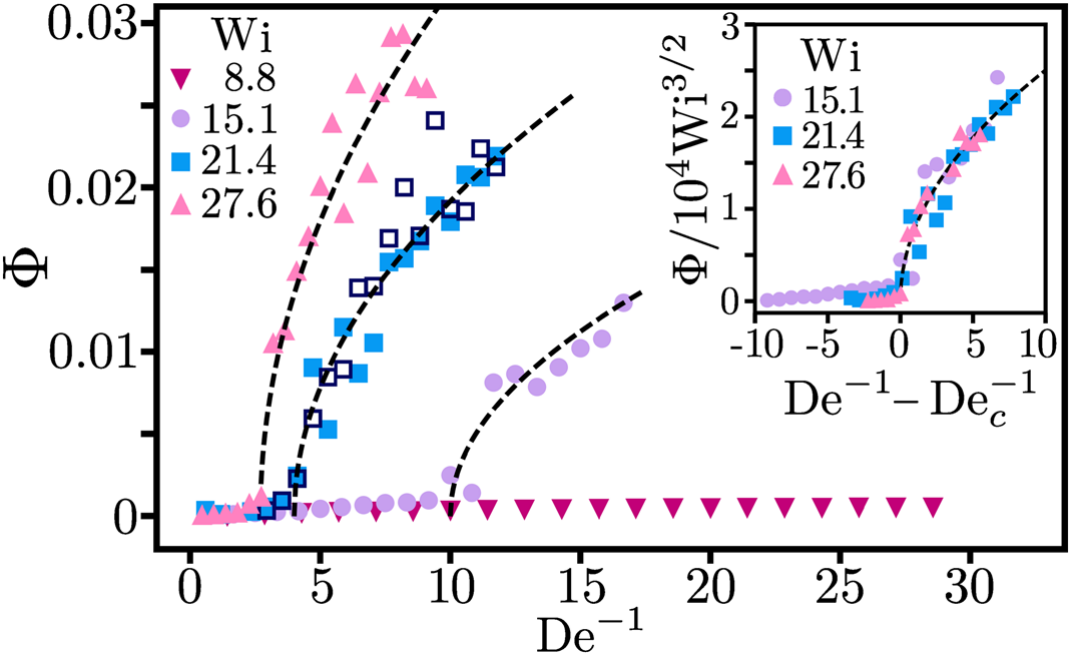
Order parameter $\Phi$ as a function of the inverse Deborah number $\mathrm {De}^{-1}=\delta /\lambda$ in the case of square wave modulations for four Weisenberg numbers. The time average of the secondary-flow strength is taken over at least 500 rotations in the turbulent regime; after the flow has been driven for 250 rotations with a constant velocity. Open blue squares: the modulated driving starts from the beginning. The dashed lines are square root fits to $\Phi \sim \sqrt{\mathrm {De}^{-1} - \mathrm {De}_c^{-1}}$. Inset: the rescaled data collapse onto a single master curve.

**Figure 4:**
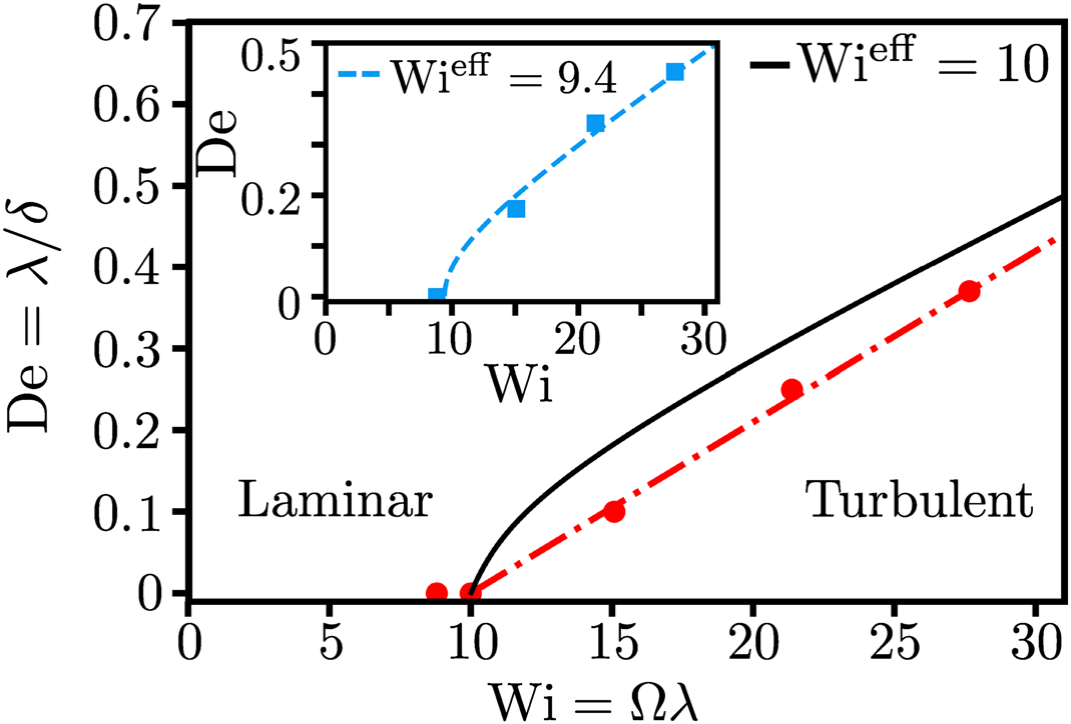
Stability diagram of the viscoelastic fluid as a function of Weissenberg number $\mathrm {Wi}=\Omega \lambda$ and Deborah number $\mathrm {De}=\lambda /\delta$ for square wave modulation. The transition between the laminar and turbulent states is demarcated by the dashed-dotted line $\mathrm {De} = 0.02 ( \mathrm {Wi} - 10)$. The critical effective $\mathrm {Wi^{eff}_c} = \mathrm {Wi}_c=10$ from Eq. (9) is indicated with the black solid line. Inset: Stability diagram for sine wave modulation. Note, for the sine wave modulation the value $\mathrm {Wi}_c = 9.4$ for $\mathrm {De} \rightarrow 0$ is an approximation. The dashed line indicating the transition $\mathrm {Wi_c^{eff}} = \mathrm {Wi}_c$ is presented in the supplemental material.

In Fig. 3 we plot the order parameter, defined as the time average of the secondary-flow strength, $\Phi = \overline{\sigma }$, versus the inverse Deborah number for different Weissenberg numbers under square wave driving. It sharply increases above a critical value $\mathrm {De}_c^{-1}$, which depends on $\mathrm {Wi}$. The transition scales as $(\mathrm {De}^{-1}- \mathrm {De}_c^{-1})^{1/2}$ implying that it is supercritical. This result is further tested by applying the modulated driving directly to the rest state (open square symbols for $\mathrm {Wi} = 21.4$ in Fig. 3). The different initial conditions do not lead to different values of $\Phi$, as is expected for a supercritical transition. We also checked that the supercritical transition occurs for sinusoidal driving. The order parameter $\Phi$ displays qualitatively similar behavior. However, the critical values $\mathrm {De}^{-1}_c$ are smaller compared to the square wave driving for the same $\mathrm {Wi}$ and $\Phi$ quickly reaches a maximum value. The results are presented in the supplemental material. Another striking feature is that the order parameter displays universal behavior around the transition. Indeed, as the inset of Fig. 3 demonstrates, all curves for different $\mathrm {Wi}$ fall on a single master curve when we normalize $\Phi$ by $\mathrm {Wi}^{3/2}$ and plot them versus $\mathrm {De}^{-1} - \mathrm {De}_c^{-1}$. To illustrate how the transition towards elastic turbulence depends on both dimensionless numbers ($\mathrm {De}$, $\mathrm {Wi}$), we have plotted the state diagram in Fig. 4 for both modulation types. It clearly demonstrates how the critical Weissenberg number, at which the elastic instability occurs, increases upon increasing the Deborah number, meaning when the frequency of the modulated driving is increased.

**Figure 5:**
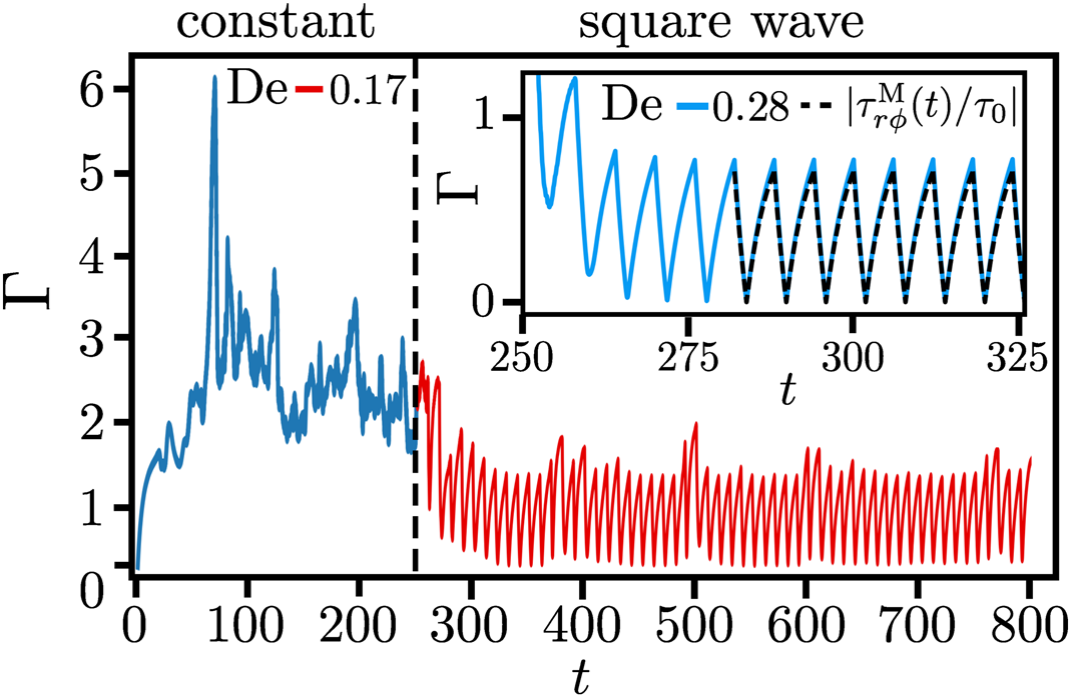
The flow resistance at the outer cylinder, $\Gamma = \left\langle \left| \tau _{r\phi }(r_o) /\tau _{r\phi }^0(r_o) \right| \right\rangle _{\phi }$, plotted versus *t* at $\mathrm {Wi}=21.4$. The square wave driving with $\mathrm {De}=0.17$ starts at $t=250 \, \mathrm {s}$. Inset: $\mathrm {De}=0.28$. The dashed line indicates the analytic result $|\tau ^{\mathrm {M}}_{r\phi }(t)/\tau _0|$, where $\tau ^{\mathrm {M}}_{r\phi }(t)$ is given by Eq. (8).

**Figure 6:**
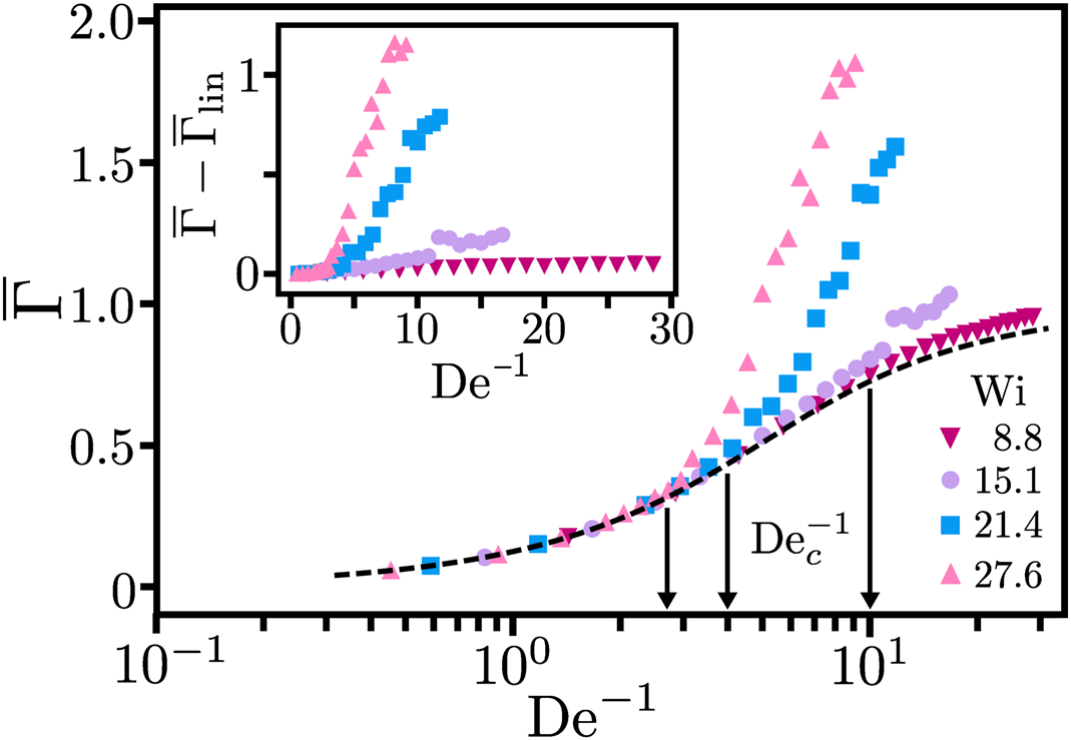
Time-averaged flow resistance or polymeric shear stress at the outer cylinder, $\overline{\Gamma }$, plotted versus the inverse Deborah number $\mathrm {De}^{-1}=\delta /\lambda$, for the same parameters as in Fig. 3. The dashed line indicates $\overline{\Gamma }_{\mathrm {lin}}$ for the stress response of the linear Oldroyd-B model. The arrows indicate $\mathrm {De}^{-1}_c$ from right to left for $\mathrm {Wi} = 15.1$, 21.4, and 27.6. Inset: $\overline{\Gamma } - \overline{\Gamma }_{\mathrm {lin}}$ versus $\mathrm {De}^{-1}$.

The elastic nature of the transition to elastic turbulence can also be monitored by the polymeric shear stress $\tau _{r\phi }$, which, when calculated at the outer cylinder, serves as an experimentally accessible measure for the flow resistance. For the steady azimuthal base flow the shear stress component becomes $\tau _{r\phi }^0 = -2 \eta _p B r^{-2}$^37^. Note that it does not depend on $\lambda$ or $\mathrm {Wi}$. Thus for the azimuthal flow the non-linear terms in the constitutive relation (1) of the polymeric stress tensor are not relevant. Now, we introduce the flow resistance using the shear stress at the outer cylinder ($r=r_o$),

7 $$\begin{aligned} \Gamma \equiv \left\langle \left| \tau _{r\phi }(r_o) /\tau _{r\phi }^0(r_o) \right| \right\rangle _{\phi } \, . \end{aligned}$$

For the steady laminar base flow, $\Gamma =1$, as defined. Under constant driving for $\mathrm {Wi}>\mathrm {Wi}_c$, elastic turbulence with a radial secondary flow develops^55^. Through the non-linear terms in Eq. (1) all polymeric stress components couple to each other and one has $\Gamma > 1$. This is illustrated in Fig. 5 until time $t=250$. Then the square-wave driving is switched on. For $\mathrm {De}=0.17 <\mathrm {De}_c$, $\Gamma$ is reduced but still reaches values above one and its time evolution is still irregular, as expected for the turbulent state. In contrast, for $\mathrm {De}=0.28 > \mathrm {De}_c$, $\Gamma$ becomes regular and can be fit well by the linear version of the Oldroyd-B model (see next paragraph). Thus the laminar state of the base flow is recovered as also indicated in Fig. 3.

We can now add some understanding for the control of elastic turbulence under modulated driving. For sufficiently large period $\delta$ or $\mathrm {De}^{-1}$, the polymer elastic stress has sufficient time to build up, generate the necessary “hoop stress”^4,35^, and thereby ultimately induce elastic turbulence (also see Movies S4–S6). However, this is no longer possible for fast switching between negative (clockwise) and positive (counter clockwise) driving. The dissolved polymers can only react with small elongations similar to the fast driving of a harmonic oscillator and the generated stress is not sufficient for elastic turbulence to occur.

To quantify this argument further, we compare the polymeric shear stress to the shear stress of the linear Oldroyd-B, which is equivalent to the Maxwell model. Note again, the linear model applies to $\tau _{r\phi }$ when calculated for the azimuthal base flow. Thus we solve $\lambda \dot{\tau }_{r \phi }^{\mathrm {M}} + \tau _{r \phi }^{\mathrm {M}} = \eta _p \dot{\gamma }(t)$ for a shear rate switching periodically between $\pm \dot{\gamma }_0$. Using the formalism of Green’s function, as detailed in the supplemental material, we arrive at

8 $$\begin{aligned} \frac{\tau _{r\phi }^{\mathrm {M}}(t)}{\tau _0} = \pm \bigg ( 1 - 2 \frac{\mathrm {e}^{-(t-t_s)/\lambda }}{1+\mathrm {e}^{-\mathrm {De}^{-1}/2}} \bigg ) \, , t_s \le t \le t_s + \delta /2 \end{aligned}$$

where ± means that the applied shear rate has switched to $\pm \dot{\gamma }_0$ at time $t_s$ and $\tau _0 = \eta _p \dot{\gamma }_0$ is the shear stress of the base flow. Now we consider the time-averaged flow resistance $\overline{\Gamma }$, with $\Gamma$ defined in Eq. (7). The corresponding quantity for the linear Oldroyd-B model can be calculated using the periodic solution from Eq. (8): $\overline{\Gamma }_{\mathrm{lin}} = \frac{2}{\delta } \int _{t_s}^{t_s+\delta /2} |\tau ^{\mathrm{M}}_{r\phi }(t)/\tau _{0|} {\mathrm{d}}t = 1 + 4\, {\mathrm{De}} \ln \left( [1+\exp (- {\mathrm{De}}^{-1}/2)]/2\right)$.

Figure 6 plots $\overline{\Gamma }$ versus $\mathrm {De}^{-1}$ for different $\mathrm {Wi}$ together with the analytic result $\overline{\Gamma }_{\mathrm {lin}}$ for the linear Oldroyd-B model as the dashed line. Below the critical $\mathrm {De}_c^{-1}$ the data fall on $\overline{\Gamma }_{\mathrm {lin}}$ clearly indicating that for sufficiently fast modulation the linear response of the laminar base flow is recovered. Above $\mathrm {De}_c^{-1}$ we observe a significant increase in the stress response $\overline{\Gamma }$ due to the turbulent flow. In contrast, for $\mathrm {Wi}=8.8 < \mathrm {Wi}_c$ the flow remains laminar and $\overline{\Gamma }\approx \overline{\Gamma }_{\mathrm{lin}}$. The slightly larger values of the numerical stress data result from the non-linear terms in the constitutive relation of the stress tensor so that the simulated flow field deviates from the base flow. Again, similar behavior is observed in the case of sinusoidal driving (presented in the supplemental material). However, $\overline{\Gamma }$ increases more rapidly with $\mathrm {De}^{-1}$ than for square-wave driving.

In the end, we motivate the solid line in Fig. 4, which approximates the transition from laminar to turbulent flow. The Weissenberg number was originally defined as the ratio of normal stress difference to shear stress^36,56^. If we calculate the time average of these stresses within the linear Oldroyd-B model for the oscillating base flow (see the supplemental material), we obtain an effective Weissenberg number

9 $$\begin{aligned} \mathrm {Wi}^{\mathrm {eff}} = \frac{\overline{\,|\tau _{rr} - \tau _{\phi \phi }|\,}}{2 \alpha \overline{\,|\tau _{r\phi }|\,}} = \frac{1 - 4\, \mathrm {De}\tanh \left( \frac{1}{4\,\mathrm {De}}\right) }{1 - 4\, \mathrm {De} \ln \left( \frac{2}{1+ \mathrm {e}^{-1/2\mathrm {De}} } \right) } \mathrm {Wi} \, . \end{aligned}$$

Here $\alpha = \dot{\gamma}/\Omega$ is a geometric constant which is introduced to obtain $\mathrm {Wi}^{\mathrm {eff}} = \mathrm {Wi}$ for constant rotation and $\overline{\dots }$ denotes the time average taken over half a period. The prefactor of $\mathrm {Wi}$ on the right-hand side decreases from 1 at $\mathrm {De} = 0$ to 0 for $\mathrm {De} \rightarrow \infty$, where stresses in the polymer cannot build up anymore to induce elastic turbulence. The solid line in Fig. 4 then follows from setting $\mathrm {Wi}^{\mathrm {eff}} = \mathrm {Wi}_c=10$, where $\mathrm {Wi}_c$ is the critical Weissenberg number for steady rotation. Our theoretical prediction qualitatively describes the transition between laminar and turbulent states but also deviates from the transition line obtained in the simulations. This indicates that the transition from laminar to turbulent flow under modulated shear is more complex. It cannot be fully treated by rescaling the critical Weissenberg number for constant rotation using time-averaged stress components.

## Discussion and conclusion

Two limitations of our approach deserve special attention. The choice of the constitutive equation, the Oldroyd-B model, and our two-dimensional geometry. A drawback of the Oldroyd-B model is the possibility of infinitely extended polymers, which is unphysical. We have checked the maximum extension of our dissolved polymers (see supplemental material) and conclude that the maximum extension of our polymers remains bounded and physical. Furthermore, the Oldroyd-B model has only a single relaxation time, whereas real polymer solutions possess a broad spectrum of relaxation times. Nevertheless, the characteristics of elastic turbulence are observed in numerical simulations of this model^16,55^, indicating that the above simplifications do not qualitatively change the physical behavior. Therefore, numerical simulations provide physical insight in the complex behavior of viscoelastic fluids.

The second limitation is the two-dimensional geometry. Unfortunately, our thorough analysis, combined with the long simulation times required, is unfeasible at the moment in three-dimensional setups. In this work we focus on instabilities in the azimuthal plane, implying non-axisymmetric modes drive the instability. In the case of $\mathrm {Re}\ll 1$, stability analysis of the Oldroyd-B fluid in a Taylor–Couette geometry with narrow gap shows that a non-axisymmetric mode governs the first instability^60^. Moreover, for increasing gap width the critical Weissenberg number of both instabilities is reduced, yet the reduction of $\mathrm {Wi_c}$ of the non-axisymmetric mode is greater^60,61^. If this is also the case for wide-gap flows, then the non-axisymmetric mode determines the instability. Furthermore, for wide-gap Taylor–Couette flow, stability analysis of the upper-convected Maxwell model shows that the most unstable modes are non-axisymmetric ribbon and spiral modes, which both exhibit a supercritical instability at sufficiently wide gaps^62^. Additionally, in experiments the aspect ratio of the Taylor–Couette cell influences the onset of the elastic instability. Due to high shear gradients created in the corner between the driving wall and the static wall, a secondary flow with curved streamlines arises in viscoelastic fluids^28^, which is influenced by the aspect ratio. As a consistency check, we have compared results from our two-dimensional simulations to results of a simulation in a three-dimensional Taylor–Couette setup (see supplemental material). We conclude that the instability observed in the three-dimensional geometry is comparable to our two-dimensional geometry.

Moreover, our results also provide insight into how elastic turbulence is reduced through passive control. For example, in Ref.^31^, geometric disorder is introduced in a microfluidic flow, which leads to a disordered local shear rate and thereby delays the occurence of elastic turbulence to larger Wi. This corresponds to spatial shear rate modulations, where increased disorder is equivalent to faster modulations. Our work demonstrates how elastic turbulence is removed under modulations of the shear rate, whether temporal or spatial, and provides a simple explanation of the physical origin.

In conclusion, by modulating the shear rate of a Taylor–Couette flow using a square- or sine-wave driving of the outer cylinder, we are able to control the onset of elastic turbulence. While at small frequencies (small Deborah numbers) irregular flow patterns are still observed in our simulations of the Oldroyd-B model, we recover the regular base flow at large frequencies beyond a critical De. Here the linear Oldroyd-B model accurately describes the rheological response of our system. Thus our work demonstrates how sensitive elastic turbulence is to oscillating shear. We consider our work to be a stepping stone for active control of elastic turbulence and hope to inspire further experimental and theoretical investigations on active open-loop or feedback control of viscoelastic fluid flow, for example, in microfluidic systems.

## Methods

All our numerical results are obtained with the open-source finite-volume solver OpenFOAM^®^ for computational fluid dynamics simulations performed on polyhedral grids^63^. We give all parameters in Si units, as required by OpenFOAM^®^, and adopt a specialised solver for viscoelastic flows called rheoTool^64,65^. The rheoTool solver has been tested for accuracy in benchmark flows and it has been shown to have second-order accuracy in space and time^65^.

Our 2D geometry consists of two coaxial cylinders and we use a mesh refinement towards the inner cylinder, where velocity gradients become larger. Figure 1b shows the grid mesh resembling a spokes wheel, which we employed, with $N_r = 100$ cells in the radial direction and $N_{\phi } = 120$ cells in the angular direction. The mesh refinement is such that the ratio of the radial grid size at the inner cylinder to the one at the outer cylinder is 10. The width $\Delta r$ of the grid cells in the radial direction is ${2.0}{\times 10^{-3}}\, r_o$ at the inner cylinder and increases to ${1.9}{\cdot 10^{-2}}\,r_o$ at the outer cylinder. The time step of the simulation is $\delta t = 10^{-5}\mathrm {s}$, where the velocity, pressure, and stress fields are extracted every 5000 steps. At the two bounding cylinders we choose the no-slip boundary condition for the velocity, zero gradient for the pressure field, and an extrapolated zero gradient for the polymeric stress field, following Ref. ^65^. We use a biconjugate gradient solver combined with a diagonal incomplete LU preconditioner (DILUP-BiCG) to solve for the components of the polymeric stress tensor and a conjugate gradient solver coupled to a diagonal incomplete Cholesky preconditioner (DIC-PCG) to solve for the velocity and pressure fields^65^. Further details on the algorithm can be found in Ref.^65^ and details on the stability of the mesh can be found in Ref.^55^.

The simulations start with the viscoelastic fluid at rest, where pressure, flow, and stress fields are uniformly zero. The following geometric parameters are chosen from the viewpoint of microfluidic settings in such a way as to set a low Reynolds number. We are interested in wide-gap flow, with a focus on microfluidic devices that could be designed for mixing. The inner cylinder at radius $r_i = 2.5 {\upmu \mathrm {m}}$ is fixed and the outer cylinder at radius $r_o=4\; r_i= 10 \upmu \mathrm {m}$ rotates with either a periodically modulated square wave with amplitude ${\Omega _0 = 2\pi \, \mathrm {s}^{-1}}$ or a sine wave with amplitude ${\Omega _0^\text {sin} = \pi ^2 \, \mathrm {s}^{-1}}$. The period of the modulation is $\delta$. The Weissenberg number is defined by averaging the rotational frequency over one period:

10 $$\begin{aligned} \mathrm {Wi}=\frac{1}{\delta }\int _{0}^{\delta } \lambda |{\Omega }|\mathrm {d}t = 2 \pi \lambda \, {\mathrm {s}^{-1}} \, . \end{aligned}$$

We adjust the Weissenberg number by varying the polymeric relaxation time $\lambda$ and we set the polymeric shear viscosity to $\eta _p=0.0015 \, {\mathrm {kg/ms}}$, the solvent shear viscosity to $\eta _s=0.001 \, {\mathrm {kg/ms}}$, and the density to $\rho =1000 \,\mathrm {kg/m^3}$. The ratio of the polymeric to the solvent viscosity is then $\beta ={\eta _p}/{\eta _s}=1.5$. The fluid flow is simulated up to a ${1000}\,\mathrm {s}$.

## Supplementary information

Supplementary Video 1.

Supplementary Video 2.

Supplementary Video 3.

Supplementary Video 4.

Supplementary Video 5.

Supplementary Video 6.

Supplementary Information.
