# Supplementary material for: Active open-loop control of elastic turbulence
Source: Sci Rep. 2020 Sep 24;10:15704. doi: 10.1038/s41598-020-72402-y (PMC7519150; doi:10.1038/s41598-020-72402-y)
Supplement: Supplementary file 7 — Supplementary Information. [file 41598_2020_72402_MOESM7_ESM.pdf]

Active open-loop control of elastic turbulence

Reinier van Buel[1,*] **and Holger Stark**[1]

[1]Technische Universität Berlin, Institute of Theoretical Physics, Hardenbergstrasse 36, 10623 Berlin, Germany
[*]r.vanbuel@tu-berlin.de

Supplemental Material :
Active open-loop control of elastic turbulence

Reinier van Buel[1,*] and Holger Stark[1]

[1]Technische Universität Berlin, Institute of Theoretical Physics, Hardenbergstrasse 36, 10623 Berlin, Germany
[*]r.vanbuel@tu-berlin.de

Here, we present the main results obtained for sine-wave modulations of the shear rate, where we discuss the secondary flow strength and the flow resistance. In section 2 and 3 we present an analytic derivation of the shear stress and the azimuthal normal stress for the laminar base flow in the Taylor-Couette geometry within the Oldroyd-B model, for square and sine-wave driving, respectively. Finally, in section 4 we define an effective Weissenberg number and use it to approximately locate the elastic instability under shear-rate modulations.

1 Sine-wave driving

In this section, we present our results when a sine-wave modulated angular velocity is applied to the outer cylinder of the Taylor-Couette cell. Thus, we drive the system with only one frequency, δ^{-1}, compared to the case of square-wave driving, for which all higher harmonics of δ^{-1} are also present. Moreover, the shear rate changes gradually with a sine wave. Nevertheless, in the following, we show that the response is nearly identical to the one for square-wave driving.

1.1 Secondary flow strength

We start again with the secondary-flow strength

$$\sigma(t) \equiv \sqrt{\langle [\mathbf{u}(\mathbf{r},t) - \mathbf{u}^0(\mathbf{r},t)]^2 \rangle_{r,\phi}} \Big/ u^0_{\max} , \tag{1}$$

where for the sinusoidal driving the base flow velocity is $\mathbf{u^0}(\mathbf{r},t) = u^0_\phi \mathbf{e}_\phi$ with $u^0_\phi = Ar + Br^{-1}$, $A = \frac{r_o^2}{r_o^2 - r_i^2}\Omega_0 \sin(2\pi t/\delta)$, $B = -\frac{r_i^2 r_o^2}{r_o^2 - r_i^2}\Omega_0 \sin(2\pi t/\delta)$ and $\langle \ldots \rangle_{r,\phi}$ denotes the spatial average over coordinates r, ϕ. Analogous to the case for square-wave driving, the secondary flow strength is clearly reduced upon applying sine-wave modulations at the outer cylinder. In Fig. 1 we plot σ versus time for 5 different values for the period of the sine-wave modulaton, $10\,\mathrm{s} \leq \delta \geq 30\,\mathrm{s}$. Similar characteristics are observed: for large periods σ exhibits irregular peaks, while for small periods σ strongly tends to zero. It also shows oscillations with the driving period δ. Finally, at low δ the flow ultimately becomes laminar.

The order parameter, the time average of the secondary-flow strength $\Phi = \overline{\sigma}$, also shows comparable behavior to the square-wave driving and scales as $\Phi \sim \sqrt{\mathrm{De}^{-1} - \mathrm{De}_c^{-1}}$ close to the transition. This is shown in Fig. 2, which plots Φ versus De^{-1} for several Wi together with the square-root fits (dashed lines). In contrast to the square-wave driving, Φ quickly reaches a maximum value for Wi=21.4 and Wi = 27.6. These findings are again independent of the initial condition as the test case for Wi = 21.4 shows, where we applied the modulated driving directly to the initial rest state (open symbols in Fig. 2). The critical inverse Deborah numbers for the different Wi, $\mathrm{De}^{-1}_{c,\sin}(\mathrm{Wi} = 15.1) = 5.8$, $\mathrm{De}^{-1}_{c,\sin}(\mathrm{Wi} = 21.4) = 2.9$, and $\mathrm{De}^{-1}_{c,\sin}(\mathrm{Wi} = 27.6) = 2.2$, shift to lower values compared to the square-wave driving and thus the transitions occur at smaller δ. A possible explanation could be that the transition is related to the larger amplitude of the modulated angular velocity, $\Omega^0_{\sin} = \pi^2 \mathrm{s}^{-1} > \Omega^0_{\mathrm{sq}} = 2\pi \mathrm{s}^{-1}$.

1.2 Flow resistance

As in the main text, the elastic nature of the transition can be illuminated by the flow resistance

$$\Gamma \equiv \langle | \tau_{r\phi}(r_o) / \tau^0_{r\phi}(r_o) | \rangle_\phi , \tag{2}$$

where we rescale the shear stress $\tau_{r\phi}(r_o)$ under constant driving with amplitude $\Omega_0 = \pi^2\,\mathrm{s}^{-1}$ with

$$\tau^0_{r\phi}(r_o) = -2\eta_p B = 2\Omega_0 \eta_p \frac{r_i^2}{r_o^2 - r_i^2} . \tag{3}$$

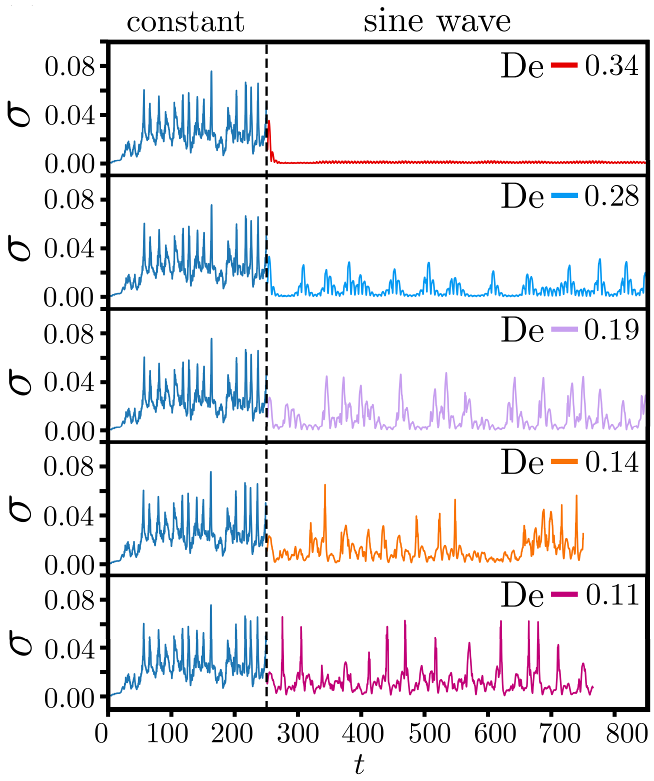

Figure 1. Secondary-flow strength σ as a function of time t for different modulation periods as indicated in the single graphs. The outer cylinder rotates with constant angular velocity $\Omega = 2\pi\,\mathrm{s}^{-1}$ for the first 250 rotations. Then the sine-wave driving with period δ and amplitude $\Omega_0 = \pi^2\,\mathrm{s}^{-1}$ is switched on. The Weissenberg number is $\mathrm{Wi} = 21.4$ resulting from the characteristic relaxation time $\lambda = 3.4\mathrm{s}$.

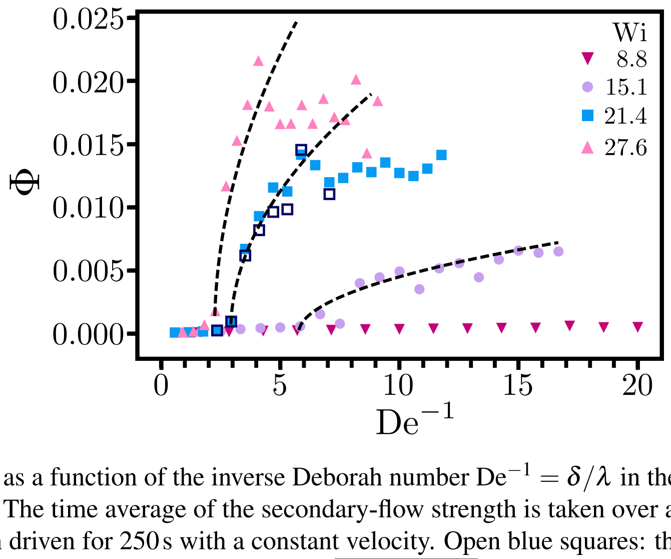

Figure 2. Order parameter Φ as a function of the inverse Deborah number $\mathrm{De}^{-1} = \delta/\lambda$ in the case of sine-wave modulations for four Weisenberg numbers. The time average of the secondary-flow strength is taken over at least 350 s in the turbulent regime; after the flow has been driven for 250 s with a constant velocity. Open blue squares: the modulated driving starts from the beginning. The dashed lines are square-root fits to $\Phi \sim \sqrt{\mathrm{De}^{-1} - \mathrm{De}_c^{-1}}$.

For the non-turbulent laminar base flow oscillating with period δ we derive in section 3, $\Gamma = \Gamma_{\text{lin}}$, where Γ_{lin} reads

$$\Gamma_{\text{lin}} = \left| \frac{2\pi \text{De} \cos(2\pi t/\delta) + \sin(2\pi t/\delta)}{1 + 4\pi^2 \text{De}^2} \right| . \tag{4}$$

As studied in the main text for square-wave driving, we start with a turbulent flow obtained for $\text{Wi} = 21.4$ for constant driving until time $t = 250\text{s}$, for which $\Gamma > 1$. Then the sine-wave driving is switched. Beyond the transition at $\text{De}^{-1} = 2.9$ or for periods larger than $\delta = 10\text{s}^{-1}$, Γ develops irregular fluctuations (see Fig. 3). They are imposed on the regular oscillations, which follow from the linear or Maxwell model in section 3. The graphs in Fig. 3 show that the time evolution of Γ becomes increasingly irregular with increasing δ and elastic turbulence is again observed.

This is further illustrated by taking the time average of Γ and plotting it versus De^{-1} in Fig. 4. Also for the sine-wave driving we compare our results to the analytic expression (38) derived in section 3 for the linear version of the Oldroyd-B model. We observe a strong increase in $\overline{\Gamma}$ beyond the transition, which is also observed from the inset of the figure, while below the transition $\overline{\Gamma} = \overline{\Gamma}_{\text{lin}}$ corresponding to the laminar the base flow.

Thus, we conclude the general behaviour is the same for square-wave and sine-wave driving. As also demonstrated in the state diagram of Fig. 4 in the main text, upon increasing the Deborah number, the transition to elastic turbulence shifts to a larger Weissenberg number Wi.

2 Oldroyd-B model in laminar flow: Square-wave modulation

In this section, we derive the stress components for the laminar flow under square-wave shear modulations using the Oldroyd-B model. For the laminar flow in our geometry the constitutive equation for the stress tensor, given in the main text, reduces to

$$\lambda \dot{\tau}_{rr} + \tau_{rr} = 0 , \tag{5}$$

$$\lambda \dot{\tau}_{r\phi} + \tau_{r\phi} = \eta_p \dot{\gamma}(t) , \tag{6}$$

$$\lambda \dot{\tau}_{\phi\phi} + \tau_{\phi\phi} = 2\lambda \dot{\gamma}(t) \tau_{r\phi} , \tag{7}$$

where $\dot{\gamma}$ is the shear rate. Considering the laminar case, where τ_{rr} starts at rest, we obtain $\tau_{rr} = 0$ for all times. In the next two sections we solve for the shear stress and the azimuthal normal stress component under square-wave driving and also obtain their time averages.

2.1 Mean polymeric shear stress

The shear stress component $\tau_{r\phi}$ can be obtained from Eq. (6), which is equivalent to the Maxwell model mentioned in the main text. Note that we skip here the superindex M in the symbol $\tau_{r\phi}$ introduced in the main text. The solution to this ODE for an arbitrary time evolution of $\dot{\gamma}(t)$ is expressed using Green's function G_c as

$$\tau_{r\phi}(t) = \int G_c(t - t') \eta_p \dot{\gamma}(t') dt' , \tag{8}$$

where the Green's function reads

$$G_c(t) = \Theta(t) \frac{1}{\lambda} \text{e}^{-t/\lambda} . \tag{9}$$

We now evaluate $\tau_{r\phi}(t)$ for the square- and sine-wave modulated shear rate used in the main text and this supplement, respectively. We also calculate the time-average of its magnitude to obtain expressions for the time-averaged flow resistance $\overline{\Gamma}$.

Here, we consider that the outer cylinder of the Taylor-Couette cell switches between clockwise and counter-clockwise constant rotation, each with a duration of $\delta/2$. Thus the shear rate at time t is either positive or negative, $\dot{\gamma}(t) = \pm\dot{\gamma}_0$. Denoting the time of the latest switching event with t_1 and times of previous events with t_n, the shear stress component of Eq. (8) becomes

$$\tau_{r\phi}(t) = \frac{\eta_p}{\lambda} \int_{-\infty}^{t} \text{e}^{-(t-t')/\lambda} \dot{\gamma}(t') dt' = \frac{\eta_p \dot{\gamma}_0}{\lambda} \left[\pm \int_{t_1}^{t} \text{e}^{-(t-t')/\lambda} dt' \pm \sum_{n=1}^{\infty} (-1)^n \int_{t_{n+1}}^{t_n} \text{e}^{-(t-t')/\lambda} dt' \right] . \tag{10}$$

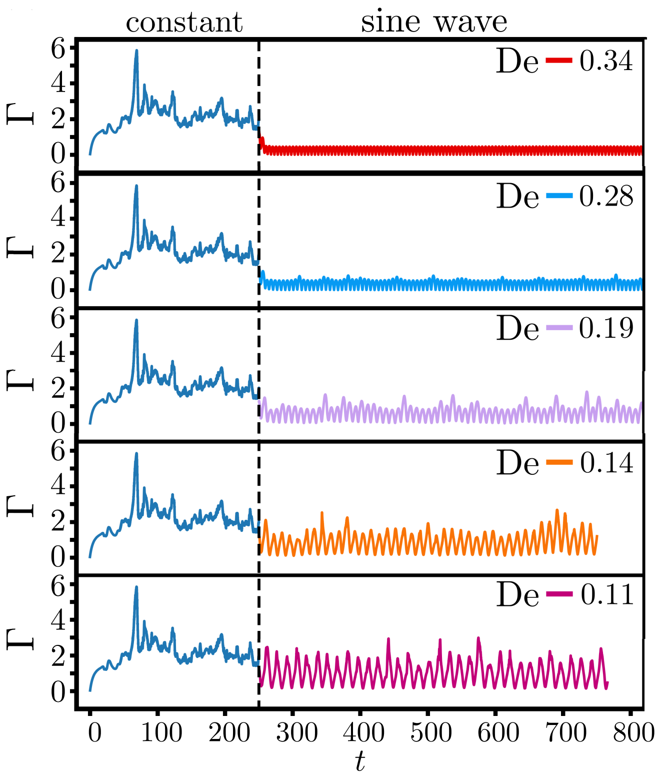

Figure 3. The flow resistance at the outer cylinder, $\Gamma = \left\langle \left| \tau_{r\phi}(r_o)/\tau^0_{r\phi}(r_o) \right| \right\rangle_\phi$, plotted versus t at $\mathrm{Wi} = 21.4$ for different driving periods as indicated in the graphs. The sine-wave driving with period δ starts at $t = 250$.

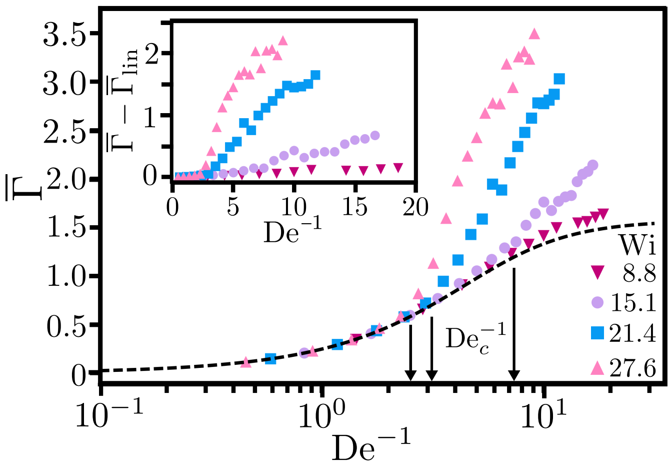

Figure 4. Time-averaged flow resistance or polymeric shear stress at the outer cylinder, $\overline{\Gamma}$, plotted versus the inverse Deborah number $\mathrm{De}^{-1} = \delta/\lambda$, for the same parameters as in Fig. 2. The dashed line indicates $\overline{\Gamma}_{\mathrm{lin}}$ for the linear stress response of a Maxwell fluid, given by Eq. (38). The arrows indicate De_c^{-1} from right to left for $\mathrm{Wi} = 15.1$, 21.4, and 27.6. Inset: $\overline{\Gamma} - \overline{\Gamma}_{\mathrm{lin}}$ versus De^{-1}.

Evaluating the integrals and restructuring the occurring sums gives

$$\tau_{r\phi}(t) = \pm\,\eta_p\dot{\gamma}_0\left[1-\mathrm{e}^{-(t-t_1)/\lambda}+\sum_{n=1}^{\infty}(-1)^n\left(\mathrm{e}^{-(t-t_n)/\lambda}-\mathrm{e}^{-(t-t_{n+1})/\lambda}\right)\right], \tag{11}$$

$$= \pm\,\eta_p\dot{\gamma}_0\left[1+\sum_{n=1}^{\infty}(-1)^n\mathrm{e}^{-(t-t_n)/\lambda}+\sum_{n=1}^{\infty}(-1)^{n+1}\mathrm{e}^{-(t-t_{n+1})/\lambda}-\mathrm{e}^{-(t-t_1)/\lambda}\right], \tag{12}$$

$$= \pm\,\eta_p\dot{\gamma}_0\left[1+\sum_{n=1}^{\infty}(-1)^n\mathrm{e}^{-(t-t_n)/\lambda}+\sum_{m=1}^{\infty}(-1)^m\mathrm{e}^{-(t-t_m)/\lambda}\right], \tag{13}$$

$$= \pm\,\eta_p\dot{\gamma}_0\left[1+2\sum_{n=1}^{\infty}(-1)^n\mathrm{e}^{-(t-t_n)/\lambda}\right]. \tag{14}$$

Now, setting $t_n = t_1-(n-1)\frac{\delta}{2}$ we obtain

$$\tau_{r\phi}(t) = \pm\eta_p\dot{\gamma}_0\left[1-2\mathrm{e}^{-(t-t_1)/\lambda}\sum_{n=0}^{\infty}\left(-\mathrm{e}^{-\delta/2\lambda}\right)^n\right] = \pm\eta_p\dot{\gamma}_0\left[1-2\mathrm{e}^{-(t-t_1)/\lambda}\frac{1}{1+\mathrm{e}^{-\delta/2\lambda}}\right], \tag{15}$$

where in the last line we have used the known expression for the sum of the geometric series. Finally, setting $\tau_0 = \eta_p\dot{\gamma}_0$ and introducing the Deborah number $\mathrm{De} = \lambda/\delta$, yields

$$\tau_{r\phi}(t) = \pm\tau_0\left[1-\frac{2\mathrm{e}^{-(t-t_1)/\lambda}}{1+\mathrm{e}^{-1/2\mathrm{De}}}\right], \quad \text{for} \quad t \geq t_1\,. \tag{16}$$

We calculate the mean polymeric stress by integrating $\tau_{r\phi}(t)$ over one full period. Since $\langle\tau_{r\phi}(t)\rangle_\delta = 0$, we consider the magnitude of the shear stress component $|\tau_{r\phi}|$ instead and integrate over half the period, from $t = t_1$ to $t = t_1+\delta/2$. The last switching event at $t = t_1$ changes the rate of strain from $\mp\dot{\gamma}_0$ to $\pm\dot{\gamma}_0$. Then the shear stress component $\tau_{r\phi}$ starts from negative/positive value at t_1 and relaxes towards a positive/negative value at $t_1+\delta/2$. In between it becomes zero at t_c. To integrate over $|\tau_{r\phi}|$, we need to know the stress values at the two boundaries of the integral but also the time t_c, which we calculate now. For $\tau_{r\phi}(t_1)$ one finds

$$\tau_{r\phi}(t_1) = \pm\tau_0\left[1-\frac{2}{1+\mathrm{e}^{-1/2\mathrm{De}}}\right] = \mp\tau_0\tanh\frac{1}{4\mathrm{De}} \tag{17}$$

and $\tau_{r\phi}(t_1+\delta/2)$ is given by

$$\tau_{r\phi}(t_1+\delta/2) = \pm\tau_0\left[1-\frac{2\mathrm{e}^{-1/2\mathrm{De}}}{1+\mathrm{e}^{-1/2\mathrm{De}}}\right] = \pm\tau_0\tanh\frac{1}{4\mathrm{De}}\,. \tag{18}$$

Now, we determine t_c from

$$\tau_{r\phi}(t_c) = \pm\tau_0\left[1-\frac{2\mathrm{e}^{-(t_c-t_1)/\lambda}}{1+\mathrm{e}^{-1/2\mathrm{De}}}\right] = 0\,, \tag{19}$$

which leads to

$$2\mathrm{e}^{-(t_c-t_1)/\lambda} = 1+\mathrm{e}^{-1/2\mathrm{De}}, \tag{20}$$

and

$$-(t_c-t_1)/\lambda = \ln\left(\frac{1+\mathrm{e}^{-1/2\mathrm{De}}}{2}\right). \tag{21}$$

With this the integral to obtain the mean shear stress component $\langle|\tau(t)_{r\phi}/\tau_0|\rangle_{\delta/2}$ can be calculated,

$$\frac{\langle|\tau_{r\phi}(t)|\rangle}{\tau_0} = \frac{2}{\delta}\int_{t_1}^{t_1+\delta/2}\left|1-\frac{2\mathrm{e}^{-(t-t_1)/\lambda}}{1+\mathrm{e}^{-1/2\mathrm{De}}}\right|dt\,, \tag{22}$$

$$= -\frac{2}{\delta}\int_{t_1}^{t_c}1-\frac{2\mathrm{e}^{-(t-t_1)/\lambda}}{1+\mathrm{e}^{-1/2\mathrm{De}}}dt+\frac{2}{\delta}\int_{t_c}^{t_1+\delta/2}1-\frac{2\mathrm{e}^{-(t-t_1)/\lambda}}{1+\mathrm{e}^{-1/2\mathrm{De}}}dt\,, \tag{23}$$

$$= \frac{2}{\delta}\left[t_1-t_c+2\lambda\frac{\mathrm{e}^{-(t_c-t_1)/\lambda}-1}{1+\mathrm{e}^{-1/2\mathrm{De}}}+\delta/2+t_1-t_c-2\lambda\frac{\mathrm{e}^{-(\delta/2)/\lambda}-\mathrm{e}^{-(t_c-t_1)/\lambda}}{1+\mathrm{e}^{-1/2\mathrm{De}}}\right], \tag{24}$$

$$= 1+\frac{4\lambda}{\delta}\left[\frac{t_1-t_c}{\lambda}+\frac{\mathrm{e}^{-(t_c-t_1)/\lambda}-1}{1+\mathrm{e}^{-1/2\mathrm{De}}}-\frac{\mathrm{e}^{-(\delta/2)/\lambda}-\mathrm{e}^{-(t_c-t_1)/\lambda}}{1+\mathrm{e}^{-1/2\mathrm{De}}}\right], \tag{25}$$

$$= 1+\frac{4\lambda}{\delta}\left[\frac{t_1-t_c}{\lambda}+\frac{2\mathrm{e}^{-(t_c-t_1)/\lambda}}{1+\mathrm{e}^{-1/2\mathrm{De}}}-\frac{1+\mathrm{e}^{-(\delta/2)/\lambda}}{1+\mathrm{e}^{-1/2\mathrm{De}}}\right]. \tag{26}$$

Inserting Eqs. (20) and (21) and using $\mathrm{De} = \lambda/\delta$ finally gives the result used in the main text,

$$\langle|\tau_{r\phi}(t)/\tau_0|\rangle = 1+4\mathrm{De}\ln\left(\frac{1+\mathrm{e}^{-1/2\mathrm{De}}}{2}\right). \tag{27}$$

2.2 Mean azimuthal normal stress.

The azimuthal stress component $\tau_{\phi\phi}$ is obtained by solving Eq.(7) using Green's function presented in Eq.(9). The time evolution of $\dot{\gamma}$ and $\tau_{r\phi}$ are periodic, which implies $\tau_{\phi\phi}$ is periodic. It follows from

$$\tau_{\phi\phi} = 2\int_{-\infty}^{t}G_c(t-t')\lambda\dot{\gamma}(t')\tau_{r\phi}(t')dt'. \tag{28}$$

Using G_c from Eq.(9), the time evolution of $\tau_{r\phi}$ from Eq. (16), defining $c = 2/(1+\mathrm{e}^{-1/2\mathrm{De}})$, and splitting the integral into integrations over one half period as before, we obtain

$$\tau_{\phi\phi} = 2\dot{\gamma}_0\tau_0\left[\int_{t_1}^{t}\mathrm{e}^{-(t-t')/\lambda}\left(1-c\,\mathrm{e}^{-(t'-t_1)/\lambda}\right)dt'+\sum_{n=1}^{\infty}\int_{t_{n+1}}^{t_n}\mathrm{e}^{-(t-t')/\lambda}\left(1-c\,\mathrm{e}^{-(t'-t_{n+1})/\lambda}\right)dt'\right]. \tag{29}$$

Performing manipulations similar to the previous subsection, we ultimately arrive at

$$\frac{\tau_{\phi\phi}}{2\lambda\dot{\gamma}\tau_0} = 1-\mathrm{e}^{-(t-t_1)/\lambda}\left(\frac{1}{2\mathrm{De}\sinh\left(\frac{1}{2\mathrm{De}}\right)}-\left[1+\tanh\left(\frac{1}{2\mathrm{De}}\right)\right]\frac{t-t_1}{\lambda}\right). \tag{30}$$

Finally, the time average over one half period gives

$$\frac{\left\langle\tau_{\phi\phi}(t)\right\rangle}{2\lambda\dot{\gamma}\tau_0} = 1-4\mathrm{De}\tanh\left(\frac{1}{4\mathrm{De}}\right). \tag{31}$$

3 Oldroyd-B model in laminar flow: Sine-wave modulation

In this section, we derive the same quantities as in the previous section, the mean polymeric shear stress and the mean azimuthal normal stress, now for the case of sinusoidal shear-rate modulations.

3.1 Mean polymeric shear stress

Here, we consider the case of sine-wave driving with $\dot{\gamma}(t) = \dot{\gamma}_0\sin(\omega t)$ in Eq.(8),

$$\tau_{r\phi}(t) = \frac{\eta_p}{\lambda}\int_{-\infty}^{t}\mathrm{e}^{-(t-t')/\lambda}\dot{\gamma}(t')dt', \tag{32}$$

and obtain for the shear stress component,

$$\tau_{r\phi}(t) = -\dot{\gamma}_0\eta_p\frac{\lambda\omega\cos(\omega t)+\sin(\omega t)}{1+\lambda^2\omega^2}, \tag{33}$$

where $\omega = 2\pi/\delta$. This is a sinusoidal variation with zeros at

$$t_n = \frac{-\arctan(2\pi\lambda/\delta)}{2\pi/\delta} + n\frac{\delta}{2}, \qquad n \in \mathbf{Z}. \tag{34}$$

The mean of the absolute polymeric shear stress component then becomes,

$$\langle|\tau_{r\phi}(t)/\tau_0|\rangle = \frac{2}{\delta}\int_{t_0}^{t1} \frac{\lambda\omega\cos(\omega t) + \sin(\omega t)}{1+\lambda^2\omega^2} dt\,, \tag{35}$$

$$\frac{\langle|\tau_{r\phi}(t)|\rangle}{\tau_0} = \frac{2}{\delta}\frac{1}{1+\lambda^2\omega^2}\left(\lambda\left[\sin\left(\frac{2\pi t_1}{\delta}\right) - \sin\left(\frac{2\pi t_0}{\delta}\right)\right] + \frac{\delta}{2\pi}\left[\cos\left(\frac{2\pi t_0}{\delta}\right) - \cos\left(\frac{2\pi t_1}{\delta}\right)\right]\right). \tag{36}$$

Now, using the identities $\sin\arctan(x) = x/\sqrt{1+x^2}$ and $\cos\arctan(x) = 1/\sqrt{1+x^2}$ the result simplifies to

$$\langle|\tau_{r\phi}(t)/\tau_0|\rangle = \frac{2}{\delta}\frac{1}{1+\lambda^2\omega^2}\left(\frac{\delta}{\pi}\sqrt{\frac{4\pi^2\lambda^2}{\delta^2}+1}\right) = \frac{2}{\pi}\frac{1}{\sqrt{\frac{4\pi^2\lambda^2}{\delta^2}+1}}. \tag{37}$$

Finally, setting $\mathrm{De} = \lambda/\delta$ leads to

$$\langle|\tau_{r\phi}(t)/\tau_0|\rangle = \frac{2}{\pi}\frac{1}{\sqrt{4\pi^2\mathrm{De}^2+1}}. \tag{38}$$

3.2 Mean azimuthal normal stress

The azimuthal stress $\tau_{\phi\phi}$ for the case of sinusoidal driving follows as in Sect. 2.2 but now with $\dot\gamma(t) = \dot\gamma_0\sin(\omega t)$. It is given by

$$\tau_{\phi\phi} = 2\lambda\int_{-\infty}^{t} G_c(t-t')\dot\gamma(t')\tau_{r\phi}(t')dt'\,. \tag{39}$$

Using Green's function from Eq. (9) and the shear stress component from Eq. (33), we obtain after some rewriting

$$\frac{\tau_{\phi\phi}}{\dot\gamma_0\tau_0} = -\int_{-\infty}^{t} \mathrm{e}^{-\frac{t-t'}{\lambda}} \frac{1+\lambda\omega\sin(2\omega t') - \cos(2\omega t')}{1+\lambda^2\omega^2} dt'\,. \tag{40}$$

Performing the integration and after some straightforward manipulations this leads to

$$\frac{\tau_{\phi\phi}}{\lambda\dot\gamma_0\tau_0} = \frac{1}{1+\lambda^2\omega^2} \tag{41}$$

where $\omega = 2\pi/\delta$. Calculating the time average over half a period gives

$$\frac{\langle\tau_{\phi\phi}(t)\rangle}{\lambda\dot\gamma_0\tau_0} = \frac{2}{\delta}\int_{t_0}^{t_1} \frac{\tau_{\phi\phi}}{\lambda\dot\gamma_0\tau_0} = \frac{1}{1+\lambda^2\omega^2}\,, \tag{42}$$

since the terms involving $\cos(2\omega t)$ and $\sin(2\omega t)$ give zero. Introducing the Deborah number, we obtain

$$\frac{\langle|\tau_{\phi\phi}(t)|\rangle}{2\lambda\dot\gamma_0\tau_0} = \frac{1}{2}\frac{1}{1+4\pi^2\mathrm{De}^2}\,, \tag{43}$$

where we used the same normalization factor as for the square-wave driving in the previous section.

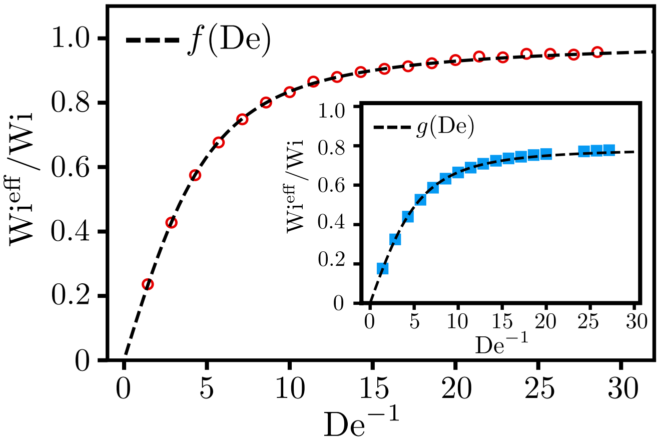

Figure 5. The effective Weissenberg number $\mathrm{Wi}^{\mathrm{eff}}$ for square wave driving normalized by the Weissenberg number Wi. The symbols are obtained from the numerical calculations at $\mathrm{Wi} = 8.8$ and the dotted black line is $f(\mathrm{De})$ given by Eq. (45). Inset: The normalized effective Weissenberg number $\mathrm{Wi}^{\mathrm{eff}}$ for sine-wave driving. The dotted black line is $g(\mathrm{De})$ given by Eq. (46).

4 Effective Weissenberg number

In this section, we define an effective Weissenberg number and introduce a criterion that approximately determines the transition to elastic turbulence under time-modulated driving. The Weissenberg number was originally defined as the ratio of normal stress difference to shear stress[1,2]. We thus introduce an effective Weissenberg number as

$$\mathrm{Wi}^{\mathrm{eff}} = \frac{1}{2\alpha}\frac{\langle|\tau_{rr}-\tau_{\phi\phi}|\rangle}{\langle|\tau_{r\phi}|\rangle} = \frac{1}{2\alpha}\frac{\langle|\tau_{\phi\phi}|\rangle}{\langle|\tau_{r\phi}|\rangle}\,, \tag{44}$$

where we used $\tau_{rr} = 0$ and $\langle\dots\rangle$ means a time average over one period as before. Note that in the main text we use a different symbol for the time average. Furthermore, we introduced the factor $\alpha = \dot{\gamma}/\Omega = 2B/(r^2\Omega)$, which links the shear rate in the base flow at radial position r to the rotational frequency, such that for constant driving the effective Weissenberg number becomes identical to the definition of Wi in the main text: $\mathrm{Wi}^{\mathrm{eff}} = \mathrm{Wi}$. Thus, the effective Weissenberg number remains a global characteristic number independent of the radial position. It is a measure for the polymeric stresses build up during time-modulated shear.

Using the time averages of the stress components, $\langle\tau_{r\phi}\rangle$ from Eq. (22) and $\langle\tau_{\phi\phi}\rangle$ from Eq. (31), we can express the effective Weissenberg number as $\mathrm{Wi}^{\mathrm{eff}} = f(\mathrm{De})\mathrm{Wi}$, where

$$f(\mathrm{De}) = \frac{1-4\,\mathrm{De}\tanh\left(\frac{1}{4\mathrm{De}}\right)}{1+4\mathrm{De}\ln\left(\frac{1+e^{-1/2\mathrm{De}}}{2}\right)} = \begin{cases} 1 & \mathrm{De}\to 0,\\ 0 & \mathrm{De}\to\infty. \end{cases} \tag{45}$$

The prefactor $f(\mathrm{De})$ is plotted in Fig. 5. It shows how the stresses in the shear modulated Oldroyd-B fluid reduce to zero with increasing modulation frequency. As an approximate critical condition for the transition to elastic turbulence, we consider now $\mathrm{Wi}_{\mathrm{c}}^{\mathrm{eff}} = f(\mathrm{De})\mathrm{Wi} = \mathrm{Wi}_c$, where $\mathrm{Wi}_c = 10$ is the critical Weissenberg number for constant driving. This defines the solid transition line in the stability diagram of Fig. 4 in the main text.

Similarly, for sine-wave driving, using Eqs. (38) and (43), we obtain for the effective Weissenberg number $\mathrm{Wi}^{\mathrm{eff}} = g(\mathrm{De})\mathrm{Wi}$, where

$$g(\mathrm{De}) = \frac{\pi}{4}\frac{1}{\sqrt{1+4\pi^2\mathrm{De}^2}} = \begin{cases} \frac{\pi}{4} & \mathrm{De}\to 0,\\ 0 & \mathrm{De}\to\infty. \end{cases} \tag{46}$$

The prefactor $g(\mathrm{De})$ is plotted in the inset of Fig. 5.

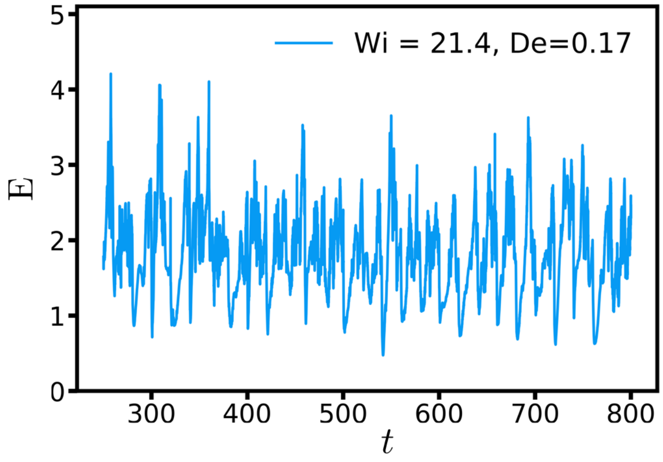

Figure 6. Normalized degree of elongation $E = \sqrt{\langle \mathrm{trC} \rangle / \langle \mathrm{trC_0} \rangle}$, where C is the conformation tensor at Wi=21.4 and De=0.17 and the conformation tensor associated with the base flow.

5 Limitations of our model

Here, we discuss some limitations of our chosen model: the finite extensibility of the dissolved polymers and the two-dimensional geometry. We first demonstrate how the polymer length remains finite. Secondly, we discuss how the instability in the same geometry develops when the full three dimensions are considered.

5.1 Polymer extension

We quantify the elongation of the polymer by the trace of the conformation tensor, which is directly proportional to the polymeric stress tensor[3]. Following Bird et al.[3] the trace of the conformation tensor C is a measure for the end-to-end distance R of the polymer $\langle \mathrm{trC} \rangle \sim \langle R^2 \rangle$. The trace of the conformation tensor in the base flow is given by

$$\langle \mathrm{trC_0} \rangle = \frac{\lambda}{\eta_p} \langle \tau^0_{\phi\phi} \rangle + \mathrm{I} \sim \langle R_0^2 \rangle , \tag{47}$$

since $\tau_{rr} = 0$ and the end-to-end distance of polymers in the base flow is denoted R_0. Now a dimensionless measure for the extension of the polymers is given by

$$\mathrm{E} = \sqrt{\frac{\langle R^2 \rangle}{\langle R_0^2 \rangle}} = \sqrt{\frac{\langle \mathrm{trC} \rangle}{\langle \mathrm{trC_0} \rangle}} , \tag{48}$$

where we normalize by the extension of the corresponding base flow $\langle \mathrm{trC_0} \rangle \sim \langle R_0^2 \rangle$. As an example, we show the time trace of E in Fig. 6 for $\mathrm{Wi} = 21.4$ and $\mathrm{De} = 0.17$ in the turbulent regime, where instead of averaging over the entire cylinder we take the maximum value. On average the extension in the turbulent regime is about twice the extension in the laminar regime. The polymers are not getting unphysically long, assuming that in the base flow they are not fully stretched.

5.2 Instability in three-dimensional Taylor-Couette geometry

The goal of our work is to demonstrate a fundamental physical effect in wall-bounded flows at low Reynolds numbers. However, as is discussed in the main text, the restricted dimensions of our geometry might influence the results. Therefore, as a consistency check, we have performed a simulation in three dimensions of a wide-gap Taylor-Couette cell at low Re. Other than the spatial dimensions we keep all the parameters the same and choose $\mathrm{Wi} = \Omega\lambda = 21.5$. The geometry consists of $N_z = 40$ mesh cells in the axial direction and is otherwise equal to our two-dimensional mesh. As is further pointed out in the main text, the aspect ratio of the flow has great influence on the stability when no-slip boundary conditions are chosen at the top and bottom walls. To limit the effect of the sharp gradients in the corner of the moving and static walls, we set the top and bottom boundaries to slip conditions. The other boundary conditions are as mentioned in the methods section in the main text.

A key element is determining the nature of the first unstable mode. As indicated in the main text, we expect the non-axisymmetric mode to be the first unstable mode in wide-gap Taylor-Couette flows. In Fig. 7 we display the side and top view snapshots of the radial component of the velocity field u_r. The base-flow component $u_r^0 = 0$ and therefore any instability arising in the velocity field is visible in the radial component. The snapshots display an instability in the azimuthal plane, while in the

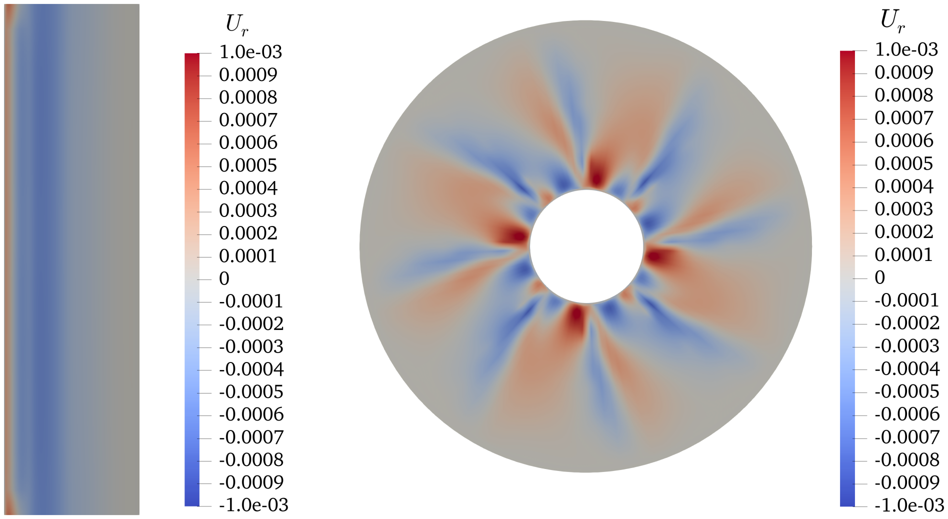

Figure 7. Snapshots of the radial component of the velocity field U_r in the three dimensional Taylor-Couette geometry. Left image is a side view at $\phi = 0$ and right is a top view at $z = h/2$, where h is the height of the cylinder. The snapshots display an instability of the base flow, where $U_r = 0$, in the azimuthal plane while the axial plane is nearly constant. Only two edge effects are visible. The snapshots are taken at $t = 10\,\mathrm{s}$.

axial plane only two edge effects are visible. These edge effects are equivalent to the described behavior above, found in Ref.[4] and due to the boundary conditions. We furthermore present snapshots of the rr-component of the stress field τ_{rr} in Fig. 8. The base flow rr-component of the stress tensor is $\tau^0_{rr} = 0$, so instabilities arising in the coupled stress components are easily identified. Figure 8 clearly shows a non-axisymmetric instability and no axi-symmetric instability.

These results strongly indicate that our two-dimensional Taylor-Couette study is sensible, also from the perspective of the instabilities expected in the three-dimensional Taylor-Couette geometry.

6 Movie legends

Movie S1. Normalized radial component of the velocity field u_r/u_{max}, where u_{max} is the maximum velocity of the base flow, under constant driving at Weissenberg number $\mathrm{Wi} = 21.4$. The radial symmetry of the base flow, where $u_r = 0$, is broken and elastic turbulence is observed.

Movie S2. Normalized radial component of the velocity field u_r/u_{max}, where u_{max} is the maximum velocity of the base flow, under square-wave modulations, with driving period $\delta = 12\,\mathrm{s}$ at Weissenberg number $\mathrm{Wi} = 21.4$ and Deborah number $\mathrm{De} = 0.28$. The radial component is significantly reduced compared to the case of constant driving. However, u_r is still irregular and the flow field still shows chaotic motion.

Movie S3. Normalized radial component of the velocity field u_r/u_{max}, where u_{max} is the maximum velocity of the base flow, under square-wave modulations, with driving period $\delta = 20\,\mathrm{s}$ at Weissenberg number $\mathrm{Wi} = 21.4$ and Deborah number $\mathrm{De} = 0.17$. The radial component is reduced compared to the case of constant driving, but the radial flow field shows strong chaotic motion comparable to the case of constant rotation.

Movie S4. Magnitude of the polymeric stress tensor $|\tau|$ under constant rotation at Weissenberg number $\mathrm{Wi} = 21.4$, which are the same conditions as Movie S1. The stress field displays a chaotic spiral-like instability and the flow is turbulent.

Movie S5. Magnitude of the polymeric stress tensor $|\tau|$ under square wave modulations, with driving period $\delta = 12\,\mathrm{s}$, at Weissenberg number $\mathrm{Wi} = 21.4$ and Deborah number $\mathrm{De} = 0.28$, which are the same conditions as Movie S2. The magnitude of the polymeric stress field is suppressed compared to the case of constant rotation and $|\tau|$ is nearly regular. After switching the direction of rotation, the stress field quickly relaxes. Afterwards it slowly builds up again and some irregular behavior is still observed.

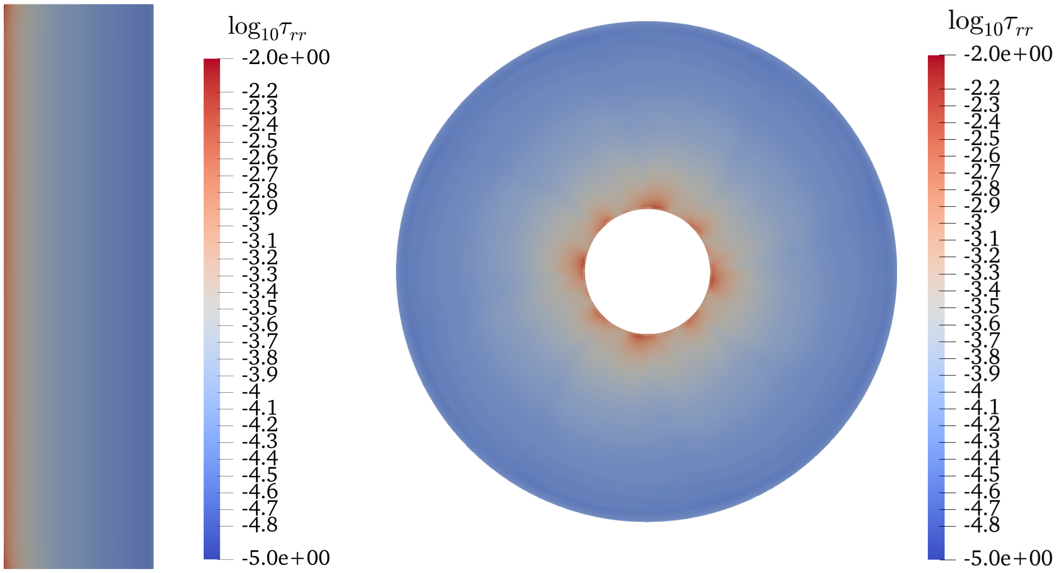

Figure 8. Snapshots of the rr-component of the stress field τ_{rr} in the three dimensional Taylor-Couette geometry. Left image is a side view at $\phi = 0$ and right is a top view at $z = h/2$, where h is the height of the cylinder. The top view shows a non-axisymmetric instability, while the side view has no axi-symmetric instability. The rr-component of the stress tensor can be easily compared to the one in the base flow, $\tau_{rr}^0 = 0$. The snapshots are taken at $t = 10\,\text{s}$.

Movie S6. Magnitude of the polymeric stress tensor $|\tau|$ under square wave modulations, with driving period $\delta = 20\,\text{s}$, at Weissenberg number $\text{Wi} = 21.4$ and Deborah number $\text{De} = 0.17$, which are the same conditions as Movie S3. The stress field displays a chaotic spiral-like instability, which is reduced compared to the case of constant rotation. After switching the direction of rotation, the stress field quickly relaxes. However, a new spiral-like instability is quickly formed. Elastic turbulence is still observed.

References

1. Dealy, J. Weissenberg and deborah numbers—their definition and use. *Rheol. Bull* **79**, 14–18 (2010).

2. Poole, R. J. The deborah and weissenberg numbers. *Rheol. Bull* **53**, 32–39 (2012).

3. Bird, R. B., Curtiss, C. F., Armstrong, R. C. & Hassager, O. In *Dynamics of Polymeric Liquids, Volume 2: Kinetic Theory* (Wiley, 1987).

4. Davoodi, M., Lerouge, S., Norouzi, M. & Poole, R. Secondary flows due to finite aspect ratio in inertialess viscoelastic taylor–couette flow. *J. Fluid Mech.* **857**, 823–850 (2018).
